# Biochemical view on: Precocious markers of cardiovascular risk and vascular damage in apparently healthy women with previous gestational diabetes

**DOI:** 10.1186/s13098-015-0023-6

**Published:** 2015-03-19

**Authors:** Huseyin Kayadibi, Erdim Sertoglu, Metin Uyanik

**Affiliations:** Adana Military Hospital, Medical Biochemistry Laboratory, Adana, 01150 Turkey; Ankara Mevki Military Hospital, Anittepe Dispensary, Biochemistry Laboratory, Ankara, Turkey; Corlu Military Hospital, Biochemistry Laboratory, Tekirdag, Turkey

**Keywords:** Gestational diabetes, Cardiovascular risk, Microangiopathy, Endothelial dysfunction, Homocysteine

## Abstract

Women diagnosed with gestational diabetes mellitus have an increased risk of developing diabetes mellitus, which is a known risk factor for cardiovascular disease and insulin resistance. In the recently published article by Zajdenverg et al., they aimed to identify endothelial dysfunction and cardiovascular risk factors in women with previous gestational diabetes mellitus. However, authors did not evaluate the role of total homocysteine, which has important effects for endothelial dysfunction. Vitamin B12 and folic acid are important vitamins since their deficiency may lead to the probable microvascular abnormalities by increasing the tHcy, which is an independent risk factor for endothelial dysfunction.

## Dear Editor

We read with great interest the recently published article by Zajdenverg et al. [1]. In this study, endothelial dysfunction and cardiovascular risk factors were aimed to identify in women with previous gestational diabetes mellitus (pGDM). In conclusion, significant differences were found in some cardiovascular risk factors when compared to others, as well as microvascular abnormalities (papillae rectification) in young women with pGDM, even with normal OGTT. However, we would like to share our thoughts and contributions to this study.

It was aimed to identify cardiovascular risk factors and microvascular abnormalities in apparently healthy women with pGDM in the original study. However, authors did not evaluate the role of total homocysteine (tHcy), which has important effects for the endothelial dysfunction. As is known, mild or moderate hyperhomocysteinemia may result from a relative deficiency of folic acid and vitamin B12. And, supplementation of the vitamins might be relevant in protection against pregnancy complications associated with elevated tHcy in GDM women [2]. Therefore, vitamin B12 and folic acid supplementation is also necessary in pregnancy and it should be better to evaluate tHcy levels to assess endothelial dysfunction in these subjects [3]. Moreover, evaluating tHcy levels may lead to get an idea for the therapy of these vitamins for the cardiovascular risk factors seen in pGDM.

In conclusion, vitamin B12 and folic acid are important vitamins since their deficiency may lead to the probable microvascular abnormalities by increasing the tHcy, which is an independent risk factor for endothelial dysfunction.

## Abbreviations

pGDM: Previous gestational diabetes mellitus
tHcy: Total homocysteine
